# Clinical, haematological, and biochemical responses of rectal oxygen-ozone administration in sheep

**DOI:** 10.1007/s11259-026-11205-4

**Published:** 2026-04-13

**Authors:** Vinicius R. C. Souza, Renan S. Carvalho, Clarisse S. Coelho, Ângela Dâmaso

**Affiliations:** 1 I-MVET, Faculty of Veterinary Medicine, Lusófona University - Lisbon University Centre, Campo Grande 376, Lisboa, 1749-024 Portugal; 2 Vila Velha University, Vila Velha - ES, Av. Comissário José Dantas de Melo, 21 - Boa Vista II, Vila Velha, 29102-920 Brazil; 3 CECAV - Centre for Veterinary and Animal Science, Faculty of Veterinary Medicine, Lusófona University - Lisbon University Center, Campo Grande 376, Lisbon, 1749-024 Portugal; 4 MED - Mediterranean Institute for Agriculture, Environment and Development, Évora University, Évora, 7006-554 Portugal

**Keywords:** Haematology, Liver enzymes, Ozone therapy, Transrectal, Sheep

## Abstract

The administration of ozone has been shown to benefit human and animal health in various areas because of its anti-inflammatory, antibacterial, antiparasitic, and antiviral effects. The effects of ozone on the health status of farm animals have not yet been described. This study hypothesises that rectal administration of an oxygen–ozone mixture in sheep is safe and causes no harm to the animals. To evaluate the clinical, haematological, and biochemical responses of sheep following rectal administration of an oxygen–ozone mixture, 10 sheep were used and divided into two experimental groups: the oxygen group (GO_2_), which was subjected to rectal insufflation with oxygen, and the ozone group (GO_2_/O_3_), which was subjected to rectal insufflation with an oxygen-ozone mixture. The treatments were carried out three times a week for four weeks. The animals were clinically assessed before and after all applications, and blood samples were taken at five time points. There were significant differences within the groups in the number of total leucocytes and the serum concentrations of GGT, total protein, albumin, and bilirubin, but the variations did not alter significantly between the treatment and control groups, except for total protein and AST. All variations, significant or not, were all kept within physiological reference limits. The results showed that treatment with the oxygen-ozone mixture did not lead to significant clinical and/or haematological changes in sheep. Therefore, ozone therapy seems to be safe to administer in sheep and should continue to be studied as an alternative treatment option for this species.

##  Introduction

 The growing challenge in veterinary medicine of using effective and innocuous therapies that are inexpensive to perform, easy to reproduce, and that reduce the use of antimicrobial agents has led to the study of ozone therapy, already widely used in human medicine (Pivotto et al. 2020). Ozone therapy is associated with antibacterial, fungicidal, viricidal, analgesic, and anti-inflammatory effects in animal species (Sciorsci et al. 2020). It acts primarily as a controlled oxidative stimulus that triggers a cascade of biochemical and immunomodulatory responses, secondary to the reaction of ozone with lipids, proteins, and antioxidants in the body, which generates reactive oxygen species (ROS) and lipid oxidation products (LOPs) (dos Santos et al. 2025). At therapeutical doses, ROS and LOPs act as secondary messengers, stimulating responses that comprise improved oxygen metabolism and availability in the body, by promoting vasodilation, neovascularisation, and tissue oxygenation; immune modulation of TNF‑α, IL‑1β, IL‑6, IL‑10, and cytokine production; and tissue regeneration by reducing oxidative stress and supporting fibroblast activity, collagen synthesis, and angiogenesis (Silva et al. 2018; dos Santos et al. 2025).

 Ozone can be delivered pure or diluted in oils, water, or blood (Bocci^2006^), and administered via intravenous, intramuscular, subcutaneous, rectal insufflation, vaginal, and intra-articular routes (Đuričić et al. 2015a), with little risk of toxicity (Bocci^2006^). A growing number of studies report ozone therapy for various pathologies, such as topical treatment of skin lesions, laminitis, facial nerve paralysis, lumbar pain, fungal, metritis and endometritis and other bacterial and viral infections, with promising results and no significant differences to antimicrobial therapies (Coelho et al. 2015; Đuričić et al. 2015b; Ouf et al. 2016; Imhof et al.^2019^; Jaramillo et al. 2020). Evidence is growing but still scarce on its effects in farm animals as an alternative to conventional therapies (Sumida and Hayashi 2022; Đuričić et al. 2015a; Samardžija 2017).

 With all this, the present study hypothesises that rectal administration of an oxygen–ozone mixture in sheep is safe and has no deleterious effects to sheep. Therefore, the aim of the study was to assess rectal ozone insufflation in ovine species and to investigate the effects of rectal oxygen–ozone administration on the clinical parameters, and on serum variables used to assess liver function and blood count in sheep.

## Materials and methods

 This research project was approved by the Ethics Committee for the Use of Animals (CEUA) at the University of Vila Velha, in Espírito Santo, Brazil (UVV-ES), under registration number 308/2014.

 Ten sheep were used, including eight females and two males. Sample size was limited to availability, but study design allowed each animal to act as its own control. The animals were clinically healthy and had been maintained under the same diet and management conditions for 30 days prior to the study. 

 The sheep were randomly divided into two experimental groups: the oxygen group (GO_2_), composed of four females and one male (average age of 2.7 ± 1.3 years; average body weight of 41.60 ± 17.33 kg), which was subjected to rectal insufflation with oxygen gas; and the ozone group (GO_2_/O_3_), composed of four females and one male (average age of 2.8 ± 1.3 years; average body weight of 39.80 ± 8.44 kg), which was subjected to rectal insufflation with the oxygen-ozone mixture.

 The calculated volume of gas to be insufflated was based on that described by Siqueira et al*.* (2025) and increased to doses above those references to account for a possible quenching effect produced by the solid and segmented faeces characteristic to ovine species and their frequency of defecation. After the rectal ampoule was emptied, gas was applied by inserting a probe into the rectum, with oxygen alone in GO_2_ and an oxygen-ozone mixture in GO_2_/O_3_.

 The experimental protocol (Coelho et al. 2015) was carried out over four weeks: Week 1: three applications of 20 µg/mL (300 mL) gas were carried out in each animal at 48-hour intervals; Week 2: the protocol was repeated (3 applications, q.48 h), changing only the concentration (25 µg/mL, 300 mL). Week 3: the concentration was maintained (25 µg/mL), and the volume was increased to 400 mL, again after 3 applications, every 48 h. Week 4: the concentration (30 µg/mL) and volume (500 mL) were increased, with 3 applications every 48 hours.

 The oxygen-ozone mixture was produced via an ozone generator (Ozone&Life®, São Paulo, Brazil - model 0&L 3.0 RM), which allows for oxygen flow rates ranging from 0 to 1.0 L/min (99.5% purity). 

 The animals were assessed daily, where heart rate (HR), respiratory rate, body temperature (BT), mucous membrane colour, and ruminal motility were recorded. Blood samples were taken from the jugular vein on day zero (prior to rectal administration) and once a week, 72 hours after the last weekly application, for a total of five samples per animal (T0, T1, T2, T3, and T4) to determine the liver profile - serum aspartate aminotransferase (AST), gamma glutamyl transferase (GGT), total protein, albumin, and bilirubin; and blood count, including red blood cells (RBC), haemoglobin concentration (Hb), globular volume (GV) and number of total leucocytes (Leuco). 

 The data were analysed via the GraphPad InStat 3.0 computerized statistical program, subjected to the Kolmogorov-Smirnov normality test and considered parametric when *p*>0.10. Subsequently, comparisons of treatment in each group (GO_2_ and GO_2_/O_3_) were performed by analysis of variance (ANOVA) followed by means comparison (Tukey test). The unpaired t-test was used for comparing GO_2_ and GO_2_/O_3_ data. A significance level of 5% was used. 

## Results

 Table1 shows the mean values and standard deviations of the values recorded in the oxygen group (GO_2_) and the ozone group (GO_2_/O_3_) for HR, BT, and blood count, serum AST, GGT, total protein, albumin, and bilirubin concentrations, as well as the *p* values for the groups’ means comparison. The parameters assessed in the physical examination were within the normal range for the species in all sheep (Seixas et al. 2021) throughout the study. Body temperature showed a significant decrease after T0 in both groups and again a significant increase at later stages of the study; no differences were found between groups. The leucocyte count showed a marked increase after T0 to the reference standards for the species. Albumin had a sharp drop in T1 to values below the reference range and increased significantly afterwards in both groups to values just above the upper limit of the reference range. Total protein also increased significantly after T0 in both groups and was significantly higher in GO_2_/O_3_ (*p*= 0.004). Serum AST activity did not change within any of the two groups but was significantly lower in GO_2_/O_3_ from the beginning of the study (*p*= 0.022). Serum GGT activity increased significantly in T1 and dropped significantly in T2 in GO_2_. This variation was also observed in GO_2_/O_3_but only a marginally significant effect. Total bilirubin had a significant increase within both groups after T0, but there were no significant differences between the groups.

**Table 1:** Mean values and standard deviations for heart rate (HR), body temperature (HT), red blood cell count (RBC), haemoglobin concentration (Hb), globular volume (VG), total leukocyte count (TLC), serum total protein, albumin, and bilirubin concentrations and serum AST and GGT activities in sheep subjected to oxygen (Group O_2_) and ozone (Group O_2_/O_3_) insufflation at time points T0, T1, T2, T3 and T4

	**Group**	**T0**	**T1**	**T2**	**T3**	**T4**	***p***
HR (bpm)	O_2_	73±8,2^a^	75±11,5^a^	74±14,3^a^	66±10,8^a^	82±15,1^a^	0,3404
	O_2_/O_3_	100±15,7^a^	83±22,9^a^	87±19,1^a^	73±15,9^a^	69±16,3^a^	0,094
BT (^o^C)	O_2_	38,9±0,2^b^	38,5±0,2^ab^	38,0±0,5^a^	38,5±0,3^ab^	39,1±0,4^b^	**0,011**
	O_2_/O_3_	39,2±0,5^a^	38,8±0,5^b^	38,5±0,5^c^	38,5±0,7^c^	39,3±0,4^a^	**0,036**
RBC (x10^6^/µL)	O_2_	9,90±1,8^a^	10,39±1,7^a^	11,29± 1,4^a^	10,55±2,0^a^	10,67±2,0^a^	0,807
	O_2_/O_3_	10,76±3,2^a^	11,46±1,9^a^	12,28±1,4^a^	11,54±2,3^a^	11,06±1,2^a^	0,831
Hb (g/dL)	O_2_	10,34±1,5^a^	10,94±1,1^a^	12,30±1,0^a^	10,82±1,5^a^	11,12±1,4^a^	0,250
	O_2_/O_3_	10,70±1,0^a^	11,96±1,4^a^	12,90±1,0^a^	11,88±1,8^a^	11,38±0,7^a^	0,112
GV (%)	O_2_	31,6 ± 5,1^a^	30,7 ± 3,2^a^	37,6 ± 3,9^a^	30,7 ± 4,1^a^	31,4±4,1^a^	0,070
	O_2_/O_3_	32,6 ±2,8^a^	33,6 ±4,0^a^	35,9±3,0^a^	33,5±5,3^a^	32,3±1,9^a^	0,563
Leuco (/µL)	O_2_	9710±3821^a^	22340±6478^b^	23860±4358^b^	22300±4363^b^	18460±3384^b^	**0,001**
	O_2_/O_3_	11190±2347^a^	22280±2456^b^	23380±4154^b^	25120±3118^b^	19460±3275^b^	**<0,0001**
Total protein (g/dL)	O_2_	5,7±0,6^a^	7,4±0,5^b^	7,5±0,6^b^	7,0±0,5^b^	7,2±0,5^b^	<0,001*
	O_2_/O_3_	5,7±1,3^a^	7,8±0,4^b^	7,8±0,4^b^	7,9±0,4^b^	7,7±0,7^b^	**<0,001**
Albumine (g/dL)	O_2_	2,8±0,4^b^	1,2±0,2^a^	3,1±0,1^b^	3,1±0,2^b^	3,1±0,2^b^	**<0,001**
	O_2_/O_3_	2,7±0,3^b^	1,2±0,3^a^	3,0±0,1^bc^	3,2±0,1^c^	3,1±0,2^c^	**<0,001**
AST (UI/L)	O_2_	136,9±57,9^a^	146,3±85,3^a^	129,5±51,9^a^	123,5±41,7^a^	120,3±33,7^a^	0,946*
	O_2_/O_3_	94,6±35,9^a^	105,6±17,0^a^	113,2±10,7^a^	103,8±15,1^a^	101,0±12,3^a^	0,678
GGT (UI/L)	O_2_	30,7±11,6^a^	41,3±6,8^b^	38,3±4,4^ab^	37,8±5,7^ab^	37,0±6,0^ab^	**0,011**
	O_2_/O_3_	34,8±6,3^a^	45,6±7,8^a^	41,0±9,8^a^	44,0±8,9^a^	36,0±12,3^a^	0,059
Total bilirubine (mg/dL)	O_2_	0,26 ± 0,06^b^	0,11 ± 0,06^a^	0,11 ± 0,06^a^	0,13 ± 0,06^a^	0,19±0,02^ab^	**0,003**
	O_2_/O_3_	0,28 ± 0,07^b^	0,09 ± 0,07^a^	0,09 ± 0,06^a^	0,11 ± 0,06^a^	0,13 ± 0,06^a^	**<0,001**

Lowercase letters on the same line denote a significant difference according to the Tukey test (*p*<0.05). The symbol (*) indicates significant differences between the control and experimental group. T0 (obtained before the experiment), T1 (obtained 72 hours after the first week of application), T2 (obtained 72 hours after the second week of application), T3 (obtained 72 hours after the third week of application), T4 (obtained 72 hours after the fourth week of application)

## Discussion

 In this study, an initial dose of 20 µg/mL was established as the oxidative preconditioning protocol, since doses of less than 10 µg/mL are considered biologically inefficient because they do not reach the therapeutic window owing to neutralization by circulating serum antioxidant enzymes (Sagai and Bocci 2011; Coelho et al. 2015). In addition, the doses selected during the first two weeks were within the range of 10 to 80 µg/mL, and higher doses of oxygen/ozone were administrated in week 3 and 4 to test the animals’ response, since ovine species defecate frequently and the faeces may reduce interface to produce and absorb the ROS and LOPs.

 The study design, with 72h-interval of sampling did not allow to assess short-term effects (6-12h) of the ozone. Still, the daily assessment identified no major changes in the clinical status of all animals. Only the average body temperature was close to the upper limit (Seixas et al. 2021) in both groups and at all assessment times, which may be related to the tropical climate of the region (daSilva and Minomo 1995) or animal restraint, which may trigger an alert reaction in animals responsible for increases in heart and respiratory rates and body temperature (Grandin and Shivley 2015).

 In general, the results observed in the physical parameters were similar to those in horses subjected to the therapeutic use of ozone via the transrectal route at concentrations of 10, 15, and 20 µg/mL (Coelho et al. 2015). Similarly, exposure of healthy horses to up to 1000 mL of the therapeutic ozone mixture by intravenous administration did not cause clinical changes in the same physical parameters mentioned above (Sciorsci et al. 2020). Several studies have reported ozone therapy in ruminants to treat different disorders, including metritis and endometritis (Đuričić et al. 2014; Maldonado et al. 2017), urovagina (Zobel et al. 2012), and retained placenta (Zobel et al. 2013; Đuričić et al. 2015a), with promising results when compared to antimicrobial therapy, but no clinical parameters have been described in these studies to allow comparisons with the results presented here.

 Leucocytosis was observed in both groups in the present study from the first rectal insufflation onwards, similar to that observed in calves subjected to ozonated autohaemoadministration (Terasaki et al. 2001). Stress could be a possible explanation for this increase, caused by handling and restraining the animals for sample collection, although these animals were used to be handled; or due to discomfort or even pain following rectal administration, as stress promotes the endogenous release of glucocorticoids and shift of leucocytes to the peripheral blood (Stockham and Scott 2025). On the other hand, HR increase, which is also a common response to stress and pain in sheep, was not observed in the present study. An inflammatory response to the ozone insufflation, either traumatic or chemical or both, must be considered to explain the leucocytosis (Siqueira et al. 2025). Another possibility is the insufflation with ozone mixture may have caused the leucocytosis as immune cells can take advantage of the oxygen availability generated by O_3_ for metabolism (Bocci^2006^). On the other hand, in horses, rectal insufflation did not promote changes in the number of leucocytes (Coelho et al. 2015), which was explained by the authors with healthy horses that underwent no clinical challenge during the experiment. Without a breakdown of the white blood cells constituents, it is not possible to further interpret our results.

 The values recorded throughout the experimental period for the biomarkers selected were within the reference range for sheep, except for albumin, whose results at T1 (average of 1.2 g/dL for GO_2_ and GO_2_/O_3_) were lower than the normal range described (Stockham and Scott 2025). The sharp drop in serum albumin is likely to correspond to an analytical error, as the animals were daily check and found sound. Also, this parameter returned to values close to the baseline values and were maintained stable with no significant changes. The significant increase seen in total protein did not go over the reference values, in any of the groups; and the variations were seen in both groups, meaning that it is unlikely that these changes were associated with the ozone administration. The lower serum AST activity in the GO_2_/O_3_ is unlikely related to the ozone treatment as this group of animals had lower AST levels from prior the ozone inoculation and the variations occurred similarly in both groups and were kept consistently with reference ranges.

 The transient increase at T1 in GGT and the drop in bilirubin can be explained by possible cholestasis (Stockham and Scott 2025), which can be a consequence of reduced food intake, possibly due to stress or discomfort. However, these values never reached the upper limits of the reference ranges, so not pathological results are reported. 

 In line with the beneficial effects of medical ozone already described in veterinary patients, such as antibacterial (Maldonado et al. 2017), reduction of chronic (Coelho et al. 2015) and acute (Rodríguez et al. 2019) inflammation, as well as the complications of its use (Jaramillo et al. 2020), this work contributes to the understanding of the effects of ozone therapy in sheep, where no major impact on the homeostasis of the animals were found during and following the rectal administration of ozone. 

 The selection of sheep as the animal model was based on the limited representation of farm animals in ozone‑therapy research, the practical ease of handling this species, and the scarcity of licensed veterinary pharmaceuticals for small ruminants, which makes them particularly relevant candidates for evaluating complementary therapeutic approaches. The absence of detectable differences between groups may reflect a limited responsiveness of sheep to ozone or, alternatively, the possibility that the ovine intestinal tract reduces the effective concentration of ozone administered via rectal insufflation, thereby diminishing its therapeutic potential. The lack of faecal analysis to characterise the local microbiota response further restricted the ability to interpret the broader health effects of ozone therapy in these animals, which represents a significant methodological limitation. The authors also acknowledge that the small sample size may have reduced the statistical power to identify treatment‑related changes.

## Conclusions

 Treatment with the oxygen-ozone mixture at increasing doses from 20 to 30 µg/mL was well tolerated by the sheep and did not induce significant changes in systemic biomarkers indicative of tissue damage. Nevertheless, given the novelty of this therapeutical approach and the limited sample size, further research employing broader dose ranges and different concentrations is warranted to assess both its therapeutic efficacy and any species-specific deleterious effects. Incorporating future perspectives involving microbiota‑focused research would provide a realistic and clinically relevant direction for subsequent work. Overall, this study highlights the need for further investigation in small ruminants and supports continued exploration of ozone as a potential integrative therapy in farm animals.

## Data Availability

No datasets were generated or analysed during the current study.
